# Assessment of a Price Index for Hospital Outpatient Department Services Using Commercial Claims Data in Massachusetts

**DOI:** 10.1001/jamahealthforum.2023.0650

**Published:** 2023-04-28

**Authors:** Hannah O. James, Katya Fonkych, Laura J. Nasuti, David I. Auerbach

**Affiliations:** 1 Massachusetts Health Policy Commission, Boston

## Abstract

This cross-sectional study assesses a market basket price index to evaluate hospital outpatient department price levels and growth.

## Introduction

Proposals for controlling health care spending have increasingly focused on prices in the hospital sector,^1^ which vary more than 3-fold nationally and often far exceed Medicare rates.^2^ In 2019, 57% of commercial health care spending occurred in hospital settings.^3^ Hospital outpatient department (HOPD) spending comprised more than half of this total and was the fastest growing category of spending from 2015 to 2019, with 22% growth in prices and 7% growth in utilization.^4^ However, evaluating price levels and growth for HOPD services is difficult, with thousands of distinct services ranging from laboratory testing to colonoscopies, and there are no obvious means to aggregate them. In this cross-sectional study, we assess a market basket price index to evaluate HOPD price levels and growth.

## Methods

We constructed an analytic file using the Massachusetts All-Payer Claims Database^5^ for residents with commercial insurance based on a procedure code encounter (same patient, date of service, Current Procedural Terminology [CPT] code) for all HOPD services (place of service: “19” or “22”), excluding encounters for patients that occurred on the same date as any emergency department or inpatient utilization. The total cost (“price”) for an HOPD encounter is the sum of professional and/or facility spending. Encounters were excluded if they were less than 20% of or more than 10 times the statewide HOPD median price for the CPT code.

We then created a 50-item Laspeyres price index defined as the aggregate sum of the average price of each item times its quantity—here, the 2018 average utilization rate (per 100 member-years) of each procedure code within the Massachusetts commercial All-Payer Claims Database population analyzed (Table). These quantities remain fixed for all units of analysis and all years to isolate price differences. The 50 services are those with the highest aggregate spending in Massachusetts in 2018. We focused on hospitals and health systems as the primary unit of analysis and imputed a price for entities with fewer than 20 encounters of a given CPT code using a price ratio for nonmissing services compared with the statewide average price. See the eMethods and eTable 1 in Supplement 1 for additional details. This study followed the STROBE reporting guidelines for cross-sectional studies.

**Table.** Summary of Hospital Outpatient Department (HOPD) Index Contents, 2018^a^

Index service	CPT procedure code(s)	Description	Utilization per 100 member-years^b^	Average price, $	Aggregate HOPD allowed amounts, $			% Of total HOPD	
					Total	Professional	Facility	Spending	Volume
1	77067	Screening mammography	6.4	290	29 772 348	6 817 080	22 955 268	1.6	1.4
2	45380	Colonoscopy with biopsy	1.0	1717	28 471 754	6 687 908	21 783 846	1.5	0.2
3	45385	Colonoscopy with removal	0.8	1879	24 181 958	6 762 967	17 418 991	1.3	0.2
4	88305	Surgical pathology (level IV)	4.7	303	22 903 575	10 022 147	12 881 428	1.2	1.0
5	99214	Outpatient visit, 25 min	7.1	184	20 992 518	12 834 622	8 157 896	1.1	1.5
6	43239	Gastrointestinal endoscopy with biopsy	0.8	1473	19 016 283	3 614 462	15 401 822	1.0	0.2
7	45378	Diagnostic colonoscopy	0.7	1573	16 606 509	2 059 321	14 547 188	0.9	0.1
8	74177	Abdominal and pelvic computed tomography with contrast	0.8	1190	15 588 994	2 382 422	13 206 572	0.8	0.2
9	93306	Echocardiogram	0.8	1133	14 669 879	1 826 691	12 843 188	0.8	0.2
10	97110	Therapeutic exercise, 15 min	6.2	139	13 884 358	4450	13 879 907	0.7	1.3
**Summary of HOPD index services**									
1-10	77067, 45380, 45385, 88305, 99214, 43239, 45378, 74177, 93306, 97110		NA	NA	206 088 176	53 012 070	153 076 106	11.1	6.3
11-20	99213, 80061, 84443, 71260, 85025, 80050, 82306, 71046, 80053, 76830		NA	NA	74 398 477	10 030 039	64 368 438	4.0	15.3
21-30	76642, 83036, 93017, 97140, 77065, 85027, 71250, 76856, 76536, 93005		NA	NA	40 156 212	4 234 585	35 921 626	2.2	6.5
31-40	80048, 76700, 97161, 77066, 77080, 82728, 73630, 82607, 84153, 86803		NA	NA	24 666 163	1 697 683	22 968 481	1.3	3.7
41-50	84439, 83970, 86850, 80076, 83735, 73502, 73030, 36415, 86900, 72100		NA	NA	14 262 417	519 526	13 742 891	0.8	5.5

Abbreviations: CPT, Current Procedural Terminology; NA, not applicable.

^a^ Data were analyzed from the Massachusetts All-Payer Claims Database in 2018.

^b^ Utilization rates are statewide rates for the commercial population included in the Massachusetts All-Payer Claims Database.

## Results

The HOPD price index accounted for 19.4% of statewide HOPD spending and 39.1% of HOPD volume in 2018 (Table). The statewide cost of the basket in 2018 was $22 922 (ie, the total amount in 2018 for the 50 services from an average hospital for 100 residents) and increased to $24 575 in 2020, a 7.2% price increase. Using the primary imputation method, there was a 2.8-fold variation in the index across hospitals and 1.5-fold variation across hospital systems. Price growth between 2018 and 2020 varied between 1.0% to 9.0% across hospital systems; there was also a positive linear correlation between price levels and growth by system, suggesting price variation increased over this period (Figure).

**Figure.**
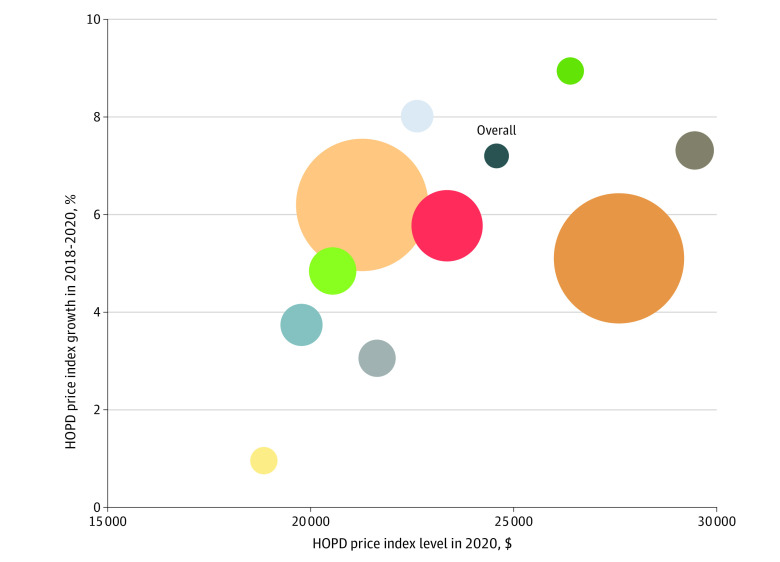
Massachusetts Hospital Outpatient Department (HOPD) Index Level in 2020 and Growth in 2018-2020 Data were analyzed from the Massachusetts All-Payer Claims Database from 2018 through 2020. Each bubble represents a health system in Massachusetts. The size of the bubble (except for the Overall data point) corresponds with the share of commercial service volume each health system provided in 2018.

## Discussion

These analyses demonstrate a novel approach to evaluating hospital prices inclusive of relevant professional and facility spending in the hospital outpatient setting. This approach enables robust price comparisons across health care organizations, payers, or states, at a point in time or across time, and can be replicated in any claims database or with newly available hospital price transparency data (along with an assumption about quantities). A limitation of this approach is that we hold volume constant to be able to isolate changes and variation in price. There may be circumstances where factoring in shifts in volume over time (eg, due to major shifts in practice patterns) may be important.

In this cross-sectional study, we identified extensive variation both in price levels and price growth across hospitals and health systems throughout Massachusetts. Opportunities to improve the value of health care spending should seek to address extensive price variation. This index is one tool that can aid in the targeting of policy efforts to identify higher-priced health care organizations and to evaluate the projected effect of potential policy changes.

## Notes

Supplement 1. **eMethods.** Key Index Components **eTable.** HOPD Index Contents, 2018

Supplement 2. Data Sharing Statement
