# Supplementary material for: Assessment of a Price Index for Hospital Outpatient Department Services Using Commercial Claims Data in Massachusetts
Source: JAMA Health Forum. 2023 Apr 28;4(4):e230650. doi: 10.1001/jamahealthforum.2023.0650 (PMC10148193; doi:10.1001/jamahealthforum.2023.0650)
Supplement: Supplement 1. — eMethods. Key Index Components eTable. HOPD Index Contents, 2018 [file jamahealthforum-e230650-s001.pdf]

Supplemental Online Content

James HO, Fonkych K, Nasuti LJ, Auerbach DI. Assessment of a price index for hospital outpatient department services using commercial claims data in Massachusetts. *JAMA Health Forum*. 2023;4(4):e230650. doi:10.1001/jamahealthforum.2023.0650

eMethods. Key Index Components
eTable. HOPD Index Contents, 2018

This supplemental material has been provided by the authors to give readers additional information about their work.

© 2023 James HO et al. *JAMA Health Forum.*

eMethods. Key Index Components

Included services

Encounters were defined as services occurring for the same person, same date of service, and same procedure code to capture the potential for both facility and professional claims billed on the same day for the same service based on the setting. As such, procedure prices and aggregate allowed amounts included any relevant facility and professional spending.

For a CPT code to be a candidate code for inclusion in the price index it had to be present with sufficient volume (at least 20 encounters) at hospital outpatient departments of at least 50 Massachusetts hospitals in 2018. 67 unique procedure codes met the above criteria. CPT codes were then ranked in descending order based on aggregate statewide HOPD spending (in 2018) and then selected the top 50 procedure codes. The set of 50 codes was chosen to include a range of typical HOPD services (e.g., visits, procedures, lab services, etc.).

Service quantities

Service quantities in the index were defined as the statewide utilization rate of each procedure code per 100 commercially insured members observed in the Massachusetts APCD in 2018. Thus, the final market basket represents expected spending per 100 commercially insured members in each year for the services in the index. The quantities are fixed, for all entities and all years, at observed levels in 2018. In accordance with a Laspeyres index, the quantities do not vary by entity, and as a result, the index values represent the cost for an identical set of services (both in type and quantity) that were rendered at hospital A versus hospital B (or system A versus system B).

Service prices

We applied an asymmetric price trimming strategy to emulate the right-skewness that is typical in cost data to reflect the price variation for commercial services. Encounters with allowed amounts that were either less 20% of the median allowed amount or greater than ten times the median allowed amount for the procedure code were excluded from analysis. After making these exclusions, we computed average service prices by taking the mean of the allowed amounts for each service for the given unit in question. We considered other approaches (e.g., excluding the top and bottom 1% of prices for each procedure code) and found that results were consistent.

Hospital Index Computation

The price index was computed for each acute care hospital in Massachusetts for each year from 2018 to 2020. Hospitals were identified in the claims data by linking to facility national provider identification (NPI) numbers.

The hospital price index is represented below where j indexes acute care hospitals and i indexes the 50 procedure codes selected for inclusion in the HOPD index ($i = 1, 2, 3, \ldots 50$). μ_{i_j} is the hospital-specific ("j-th") average price for procedure code i, and k_i represents the statewide utilization rate for procedure code i. $\sum$ signifies that we sum up the product of the hospital-specific average price μ_{i_j} and the statewide utilization rate k_i for each of the 50 index services separately by hospital to calculate the total value of the price index for each hospital:

$$HOPD\ Price\ Index_j = \sum_{i=1}^{50} \mu_{i_j} * k_i$$

© 2023 James HO et al. *JAMA Health Forum.*

Computing the Price Index for Other Units of Analysis

Hospital system: Hospital systems were defined as a group of hospitals that are affiliated. We rely on public information from the Center for Health Information and Analysis to describe the organization of acute care hospitals into hospital systems in Massachusetts during our study period.[1]

To compute the index at the level of the hospital system, we adapt the hospital-specific approach, by calculating the mean service price at the level of the hospital system (rather than the individual hospital). The procedure-specific statewide utilization rates are still employed, and the same set of services (as in the hospital index) are used across all hospital systems.

The formula for the calculation of the price index is represented below where c indexes hospital systems, i indexes the 50 procedure codes selected for inclusion in the HOPD index. μ_{i_c}represents the hospital system-specific ("c-th") average price for procedure code i, and k_i represents the statewide utilization rate for procedure code i. $\sum$ signifies that we sum up the product of the hospital system-specific average price μ_{i_c} and the statewide utilization rate k_i for each of the 50 index services separately by hospital system to calculate the total value of the price index for each hospital system:

$$HOPD\ Price\ Index_c = \sum_{i=1}^{50} \mu_{i_c} * k_i$$

Statewide: To compute the index at the level of a state, the calculation is a sum of the average statewide price for each procedure code i multiplied by the statewide utilization rate k_i for each procedure code i. $\sum$ signifies that we sum up the product of the statewide average price μ_i and the statewide utilization rate k_i for each of the 50 index services to calculate the total value of the price index for the state:

$$HOPD\ Price\ Index_{state} = \sum_{i=1}^{50} \mu_i * k_i$$

Methods for Imputation of Incomplete Index Components

In 2018, 26 hospitals had sufficient volume (at least 20 encounters) for all 50 services in the HOPD index. The remaining hospitals had at least one missing service with insufficient volume. We examined the following two imputation methods to impute average hospital-specific prices in such cases and present primary results using Method 1.

Method 1: Imputation using price ratios for non-missing services at a given hospital

This method involved the following steps:

1) For each non-missing service, we calculated the ratio of the hospital's average procedure price and the statewide average price. For example, a hospital with an average price of procedure code X that is 10 percent higher than the statewide average would have a value of 1.1 for this ratio.
2) Then we calculated the simple average of the ratios for all non-missing services. For example, if a hospital had two missing codes and ratios of 1.1 and 1.0 for the two codes, this average of ratios would be 1.05.

---

[1] Available here: https://www.chiamass.gov/assets/docs/r/hospital-profiles/2020/FY20-Hospital-Health-Systems-Technical-Appendix.pdf

© 2023 James HO et al. *JAMA Health Forum.*

3) Finally, we multiplied the statewide average price for the missing service by the ratio in step 2 to estimate a hospital-specific predicted price for a missing service. Effectively, if the hospital had provided a service, what we expect that price would have been, assuming pricing consistent with all other services.

Method 2: Imputation using statewide average prices
This imputation method directly substitutes the statewide average price for a procedure code in which a hospital does not have sufficient volume of that code to calculate its own average price.

This method is more conservative and biases a hospital's price toward the statewide average (i.e., high-priced hospitals will appear somewhat lower-priced than they likely would be and vice-versa) but is computationally simple and reduces potential volatility in the index where specific circumstances may explain why hospitals might have insufficient volume of a given procedure.

Comparison of Imputation Methods
We found that while these ratios vary somewhat by procedure for a given hospital, they tend to be relatively consistent. It is unusual for a hospital to have some prices far above the statewide mean and some far below. For example, it is more typical for a hospital with average prices 20% below the statewide mean to have individual procedure prices that vary between roughly 10% to 30% below the statewide mean.

The following exhibit illustrates the difference between HOPD index in 2019 estimated using the two methods described above, showing a selection of hospitals with at least $100 difference in the price indices.

© 2023 James HO et al. *JAMA Health Forum.*

Exhibit 1: Comparison of HOPD index levels with different imputation methods, 2019

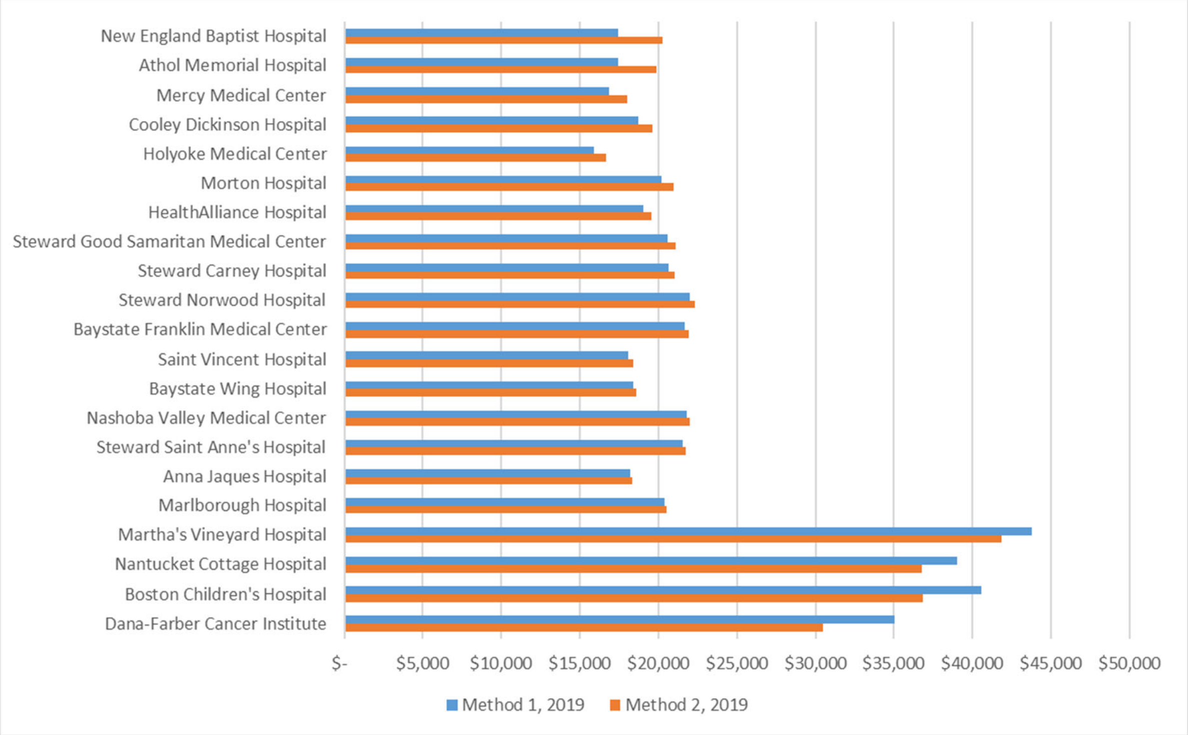

As noted above, higher-priced hospitals have a higher index value under Method 1 and lower-priced hospitals have a lower index value, whereas in Method 2, index values are compressed towards the statewide average, thus masking true price variation for non-missing services.

Complete Case Analysis for Hospitals with Non-Missing Services

Twenty hospitals have complete information for all index services (no missing index services due to insufficient volume), and do not require imputation for the calculation of the price index. Using this subset of hospitals, we performed a sensitivity analysis to evaluate varying sizes of the basket where we restrict to the top 10, 20, 30...etc. services in the basket based on statewide aggregate spending. We find high correlations across all permutations evaluated in terms of the basket size, and therefore find that the index is robust to the number of services included in it (Exhibit 2).

Exhibit 2. Pearson's correlation of hospital index results among hospitals with complete cases (n=20 hospitals), 2020

	services=10	services=20	services=30	services=40	services=50
services=10	1				
services=20	0.9564	1			
services=30	0.9564	0.9925	1		
services=40	0.9549	0.9865	0.997	1	
services=50	0.9308	0.9654	0.9774	0.9835	1

Assessing Validity of Empirically Calculated Average Prices

Since 2021, CMS has required hospitals to publish price data for "shoppable" services commonly provided by the hospital. We compared reported data from four Massachusetts

© 2023 James HO et al. *JAMA Health Forum.*

hospitals and for four major payers in each hospital (Blue Cross Blue Shield, Tufts Health Plan, Harvard Pilgrim Health Care, and Allways) to validate the facility prices we observed in the MA APCD that are inputs in the HOPD index. In a comparison of hospital-reported 2022 facility prices (the only year available at the time of study) and the median 2020 MA APCD facility prices we found that they were generally concordant (Exhibit 3)

Exhibit 3. Comparison of HOPD Index Computed Using 2022 Hospital Price Transparency Data and 2020 MA APCD Prices

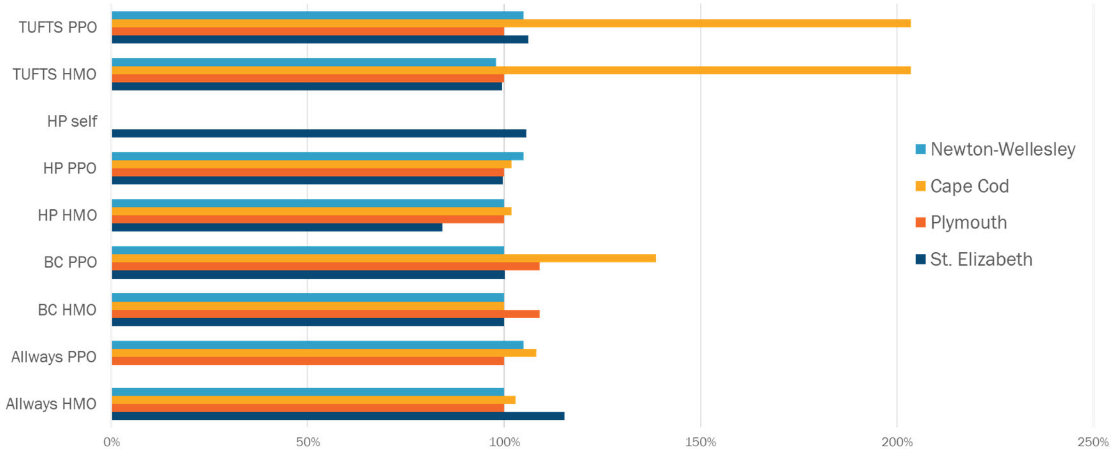

The numerator for this measure is the index value computed for the selected hospitals using 2022 hospital price transparency data (facility prices only), while the denominator is the index value computed using 2020 MA APCD facility prices only. 100% indicates that the index values are consistent. A value greater than 100% indicates the index calculated using the 2022 price transparency data exceeds the index calculated using the 2020 MA APCD. We might expect to see this across some hospitals where contracting changes occurred between 2020 and 2022 that increase prices over time, but the index values remain largely consistent.

© 2023 James HO et al. *JAMA Health Forum.*

eTable. HOPD Index Contents, 2018

CPT	Description	Utilization per 100 member years	Average price ($)	Aggregate HOPD Allowed Amounts ($)			Percent of Total HOPD Spend	Running Percent Sum - Spending	Percent of Total HOPD Volume	Running Percent Sum - Volume	Encounters
				Total	Professional	Facility					
77067	SCREENING MAMMOGRAPHY	6.4	290	29,772,348	6,817,080	22,955,268	1.6%	1.6%	1.4%	1.4%	102,540
45380	COLONOSCOPY W/BIOPSY SINGLE/MULTIPLE	1.0	1,717	28,471,754	6,687,908	21,783,846	1.5%	3.1%	0.2%	1.6%	16,585
45385	COLSC FLX PROX SPLENIC FLXR RMVL LES SNARE TQ	0.8	1,879	24,181,958	6,762,967	17,418,991	1.3%	4.5%	0.2%	1.8%	12,868
88305	LEVEL IV SURG PATHOLOGY GROSS&MICROSCOPIC EXAM	4.7	303	22,903,575	10,022,147	12,881,428	1.2%	5.7%	1.0%	2.8%	75,498
99214	OFFICE OUTPATIENT VISIT 25 MINUTES	7.1	184	20,992,518	12,834,622	8,157,896	1.1%	6.8%	1.5%	4.3%	114,061
43239	EDG TRANSORAL BIOPSY SINGLE/MULTIPLE	0.8	1,473	19,016,283	3,614,462	15,401,822	1.0%	7.8%	0.2%	4.5%	12,907
45378	COLONOSCOPY FLX DX W/WO COLLJ SPECIMENS	0.7	1,573	16,606,509	2,059,321	14,547,188	0.9%	8.7%	0.1%	4.6%	10,554
74177	CT ABDOMEN & PELVIS W/CONTRAST MATERIAL	0.8	1,190	15,588,994	2,382,422	13,206,572	0.8%	9.6%	0.2%	4.8%	13,097
93306	ECHO TTHRC R-T 2D W/WOM-MODE COMPL SPEC&COLR D	0.8	1,133	14,669,879	1,826,691	12,843,188	0.8%	10.4%	0.2%	5.0%	12,947
97110	THERAPEUTIC PX 1/> AREAS EACH 15 MIN EXERCISES	6.2	139	13,884,358	4,450	13,879,907	0.7%	11.1%	1.3%	6.3%	99,677

© 2023 James HO et al. *JAMA Health Forum.*

99213	OFFICE OUTPATIENT VISIT 15 MINUTES	7.1	117	13,284,979	6,584,053	6,700,926	0.7%	11.8%	1.5%	7.9%	113,582
80061	LIPID PANEL	16.1	33	8,505,098	1,945	8,503,153	0.5%	12.3%	3.5%	11.3%	257,009
84443	ASSAY OF THYROID STIMULATING HORMONE TSH	9.7	54	8,311,896	1,206	8,310,690	0.4%	12.8%	2.1%	13.4%	154,886
71260	CT THORAX W/CONTRAST MATERIAL	0.7	633	7,097,181	1,427,622	5,669,558	0.4%	13.1%	0.2%	13.5%	11,211
85025	BLOOD COUNT COMPLETE AUTO&AUTO DIFRNTL WBC	15.3	28	6,860,677	1,786	6,858,891	0.4%	13.5%	3.3%	16.8%	245,306
80050	GENERAL HEALTH PANEL	3.1	132	6,454,104	130	6,453,974	0.3%	13.9%	0.7%	17.5%	49,041
82306	25 HYDROXY INCLUDES FRACTIONS IF PERFORMED	4.3	88	6,095,128	194	6,094,935	0.3%	14.2%	0.9%	18.4%	68,992
71046	RADIOLOGIC EXAMINATION, CHEST; 2 VIEWS	2.5	153	6,088,260	777,296	5,310,964	0.3%	14.5%	0.5%	19.0%	39,727
80053	COMPREHENSIVE METABOLIC PANEL	11.3	33	5,997,533	431	5,997,101	0.3%	14.8%	2.4%	21.4%	181,132
76830	ULTRASOUND TRANSVAGINAL	1.2	286	5,703,622	1,235,376	4,468,246	0.3%	15.1%	0.3%	21.7%	19,912
76642	US BREAST, UNILATERAL REAL TIME IMGE DOCM	1.3	251	5,080,322	1,248,409	3,831,913	0.3%	15.4%	0.3%	21.9%	20,266
83036	HEMOGLOBIN GLYCOSYLATED A1C	9.2	30	4,501,342	8,685	4,492,657	0.2%	15.7%	2.0%	23.9%	147,651
93017	CV STRS TST XERS&/OR RX CONT ECG TRCG ONLY	0.6	495	4,411,745	-	4,411,745	0.2%	15.9%	0.1%	24.0%	8,909
97140	MANUAL THERAPY TQS 1/> REGIONS	2.8	98	4,363,830	1,744	4,362,085	0.2%	16.1%	0.6%	24.6%	44,304

© 2023 James HO et al. *JAMA Health Forum.*

	EACH 15 MINUTES										
77065	DIAGNOSTIC MAMMOGRAPHY, UNILATERAL, INCLUDING CAD	0.9	278	4,207,065	899,615	3,307,450	0.2%	16.4%	0.2%	24.8%	15,118
85027	BLOOD COUNT COMPLETE AUTOMATED	11.0	21	3,742,764	163	3,742,601	0.2%	16.6%	2.4%	27.2%	175,081
71250	CT THORAX W/O CONTRAST MATERIAL	0.5	474	3,740,341	813,979	2,926,362	0.2%	16.8%	0.1%	27.3%	7,898
76856	US PELVIC NONOBSTETRIC REAL-TIME IMAGE COMPLETE	0.7	285	3,404,244	682,849	2,721,395	0.2%	17.0%	0.2%	27.5%	11,952
76536	US SOFT TISSUE HEAD & NECK REAL TIME IMGE DOCM	0.7	308	3,387,721	579,014	2,808,706	0.2%	17.1%	0.1%	27.6%	10,996
93005	ECG ROUTINE ECG W/LEAST 12 LDS TRCG ONLY W/O I&R	2.4	86	3,316,837	126	3,316,711	0.2%	17.3%	0.5%	28.1%	38,611
80048	BASIC METABOLIC PANEL CALCIUM TOTAL	7.8	26	3,192,045	166	3,191,879	0.2%	17.5%	1.7%	29.8%	124,551
76700	US ABDOMINAL REAL TIME W/IMAGE DOCUMENTATION	0.6	354	3,146,437	692,145	2,454,292	0.2%	17.7%	0.1%	29.9%	8,881
97161	PHYSICAL THERAPY EVALUATION, LOW COMPLEXITY	0.9	198	2,771,022	90	2,770,932	0.1%	17.8%	0.2%	30.1%	14,010
77066	DIAGNOSTIC MAMMOGRAPHY, BILATERAL,	0.5	357	2,782,313	527,406	2,254,907	0.2%	18.0%	0.1%	30.2%	7,794

© 2023 James HO et al. *JAMA Health Forum.*

	INCLUDING CAD									
77080	DXA BONE DENSITY STUDY 1/> SITES AXIAL SKEL	0.7	219	2,365,676	200,313	2,165,363	0.1%	18.1%	0.1%	30.4%	10,803
82728	ASSAY OF FERRITIN	3.1	45	2,270,133	168	2,269,965	0.1%	18.2%	0.7%	31.0%	50,215
73630	RADEX FOOT COMPLETE MINIMUM 3 VIEWS	0.9	150	2,256,434	277,059	1,979,375	0.1%	18.3%	0.2%	31.2%	15,079
82607	CYANOCOBALAMIN VITAMIN B-12	2.8	46	2,034,715	152	2,034,564	0.1%	18.4%	0.6%	31.8%	44,669
84153	ASSAY OF PROSTATE SPECIFIC ANTIGEN TOTAL	2.3	54	1,980,666	163	1,980,503	0.1%	18.5%	0.5%	32.3%	36,495
86803	HEPATITIS C ANTIBODY	2.7	43	1,866,722	20	1,866,702	0.1%	18.6%	0.6%	32.9%	43,899
84439	ASSAY OF FREE THYROXINE	3.7	31	1,825,319	46	1,825,274	0.1%	18.7%	0.8%	33.7%	59,568
83970	ASSAY OF PARATHORMONE	0.7	137	1,627,749	38	1,627,711	0.1%	18.8%	0.2%	33.9%	11,901
86850	ANTIBODY SCREEN RBC EACH SERUM TECHNIQUE	1.9	51	1,544,977	10	1,544,967	0.1%	18.9%	0.4%	34.3%	30,150
80076	HEPATIC FUNCTION PANEL	3.4	26	1,407,664	-	1,407,664	0.1%	19.0%	0.7%	35.0%	53,625
83735	ASSAY OF MAGNESIUM	3.7	23	1,366,109	17	1,366,092	0.1%	19.1%	0.8%	35.8%	59,452
73502	RADEX HIP W/WO PELVIS, 2/3 VIEWS	0.5	172	1,364,734	167,313	1,197,421	0.1%	19.1%	0.1%	35.9%	7,945
73030	RADEX SHOULDER COMPLETE MINIMUM 2 VIEWS	0.6	145	1,353,228	175,953	1,177,274	0.1%	19.2%	0.1%	36.0%	9,351
36415	COLLECTION VENOUS BLOOD	11.9	7	1,272,703	13,069	1,259,634	0.1%	19.3%	2.6%	38.6%	189,991

© 2023 James HO et al. *JAMA Health Forum.*

	VENIPUNCTUR E										
869 00	BLOOD TYPING ABO	1.9	40	1,249,8 74	8	1,249,8 66	0.1 %	19.3 %	0.4 %	39.0 %	30,956
721 00	RADEX SPINE LUMBOSACRA L 2/3 VIEWS	0.5	154	1,250,0 59	163,071	1,086,9 88	0.1 %	19.4 %	0.1 %	39.1 %	8,117

© 2023 James HO et al. *JAMA Health Forum.*
