# Supplementary material for: Assessment of a Price Index for Hospital Outpatient Department Services Using Commercial Claims Data in Massachusetts
Source: JAMA Health Forum. 2023 Apr 28;4(4):e230650. doi: 10.1001/jamahealthforum.2023.0650 (PMC10148193; doi:10.1001/jamahealthforum.2023.0650)
Supplement: Supplement 2. — Data Sharing Statement [file jamahealthforum-e230650-s002.pdf]

Data Sharing Statement

James. Assessment of a Price Index for Hospital Outpatient Department Services Using Commercial Claims Data in Massachusetts. *JAMA Health Forum*. Published April 28, 2023. doi:10.1001/jamahealthforum.2023.0650

Data
Data available: No

Additional Information
Explanation for why data not available: The data use agreement for the MA APCD data does not permit us to make the underlying data publicly available. However, individuals interested in acquiring this data for their own work can inquire with the Center for Health Information and Analysis, which is responsible for disseminating these data.
